# Exploiting Paradoxical Activation of Oncogenic MAPK Signaling by Targeting Mitochondria to Sensitize *NRAS* Mutant-Melanoma to Vemurafenib

**DOI:** 10.3390/ijms26062675

**Published:** 2025-03-16

**Authors:** Laura Francisca Leite do Prado-Souza, Letícia Silva Ferraz, Tharcísio Citrangulo Tortelli, César Augusto João Ribeiro, Danilo Trabuco do Amaral, Denise Costa Arruda, Érica Aparecida de Oliveira, Roger Chammas, Silvya Stuchi Maria-Engler, Tiago Rodrigues

**Affiliations:** 1 Center for Natural and Human Sciences (CCNH), Federal University of ABC (UFABC), Santo Andre, Sao Paulo 09210-580, Brazil; laura.francisca@ufabc.edu.br (L.F.L.d.P.-S.); leticia.conconi@ufabc.edu.br (L.S.F.); cesar.ribeiro@ufabc.edu.br (C.A.J.R.); danilo.trabuco@ufabc.edu.br (D.T.d.A.); 2 Center for Translational Research in Oncology (LIM24), Cancer Institute of the State of Sao Paulo (ICESP), Clinical Hospital of the University of Sao Paulo Medical School (HCFMUSP), Sao Paulo 01246-000, Brazil; tharcisio.junior@hc.fm.usp.br (T.C.T.J.); rchammas@usp.br (R.C.); 3 Comprehensive Center for Precision Oncology, University of São Paulo, Sao Paulo 05508-220, Brazil; 4 Integrated Biotechnology Nucleus (NIB), University of Mogi das Cruzes (UMC), Mogi das Cruzes, Sao Paulo 08780-911, Brazil; denisearruda@umc.br; 5 Centre for Evolution and Cancer, The Institute of Cancer Research, London SM2 5NG, UK; erica.oliveira@icr.ac.uk; 6 Department of Clinical and Toxicological Analysis, Faculty of Pharmaceutical Sciences, University of Sao Paulo, Sao Paulo 05508-220, Brazil; silvya@usp.br

**Keywords:** cancer, *NRAS*, targeted therapy, mitochondrial dynamics, mdivi-1, oxidative phosphorylation

## Abstract

Vemurafenib is a BRAF (rapidly accelerated fibrosarcoma B-type)-targeted therapy used to treat patients with advanced, unresectable melanoma. It inhibits the MAPK (mitogen-activated protein kinase)/ERK (extracellular signal-regulated kinase) pathway and tumor proliferation in BRAF^V600E^-mutated melanoma cells. Resistance to vemurafenib has been reported in melanoma patients due to secondary *NRAS* (neuroblastoma RAS viral oncogene homolog) mutations, which lead to paradoxical MAPK pathway activation and tumor proliferation. However, the impact of this paradoxical activation on mitochondrial dynamics and function in *NRAS*-mutated melanoma is unclear. Here, we investigated the effects of vemurafenib on NRAS^Q61R^-mutated melanoma cells, focusing on mitochondrial dynamics and function. As expected, vemurafenib did not exhibit cytotoxicity in SK-MEL-147 NRAS^Q61R^-mutated melanoma cells, even after 72 h of incubation. However, it significantly enhanced the MAPK/ERK signaling through paradoxical activation, accompanied by decreased expression of mitochondrial fusion proteins and activation of the fission protein DRP1 (dynamin-related protein 1), leading to small, rounded mitochondrial morphology. These observations were corroborated by transcriptome data obtained from *NRAS*-mutated melanoma patients, showing *MFN1* (mitofusin 1) and *OPA1* (optic atrophy 1) downregulation and *DNM1L* (DRP1 gene) upregulation. Interestingly, inhibition of mitochondrial fission with mdivi-1 or modulation of oxidative phosphorylation via respiratory chain inhibition or uncoupling significantly sensitized NRAS^Q61R^-mutated melanoma cells to vemurafenib. Despite vemurafenib’s low cytotoxicity in *NRAS*-mutated melanoma, targeting mitochondrial dynamics and/or oxidative phosphorylation may offer a promising strategy for combined therapy.

## 1. Introduction

Melanoma is a type of skin cancer originating from melanocytes, characterized by genetic mutations and epigenetic alterations that drive tumor progression, including neoangiogenesis, uncontrolled proliferation, tissue invasion, immune evasion, and resistance to cell death [1]. Melanoma is reported as the fifth most common type of cancer worldwide [2] with estimated global incidence rates of 2.8 to 3.1 per 100,000 people [3]. Although melanoma accounts for only 1% of all skin cancer cases globally, it is responsible for 80% of skin cancer-related deaths due to its aggressive nature [4].

Mutations in *BRAF* (rapidly accelerated fibrosarcoma B-type) and *NRAS* (neuroblastoma RAS viral oncogene homolog) are the most common genetic drivers of melanoma, with BRAF^V600E^ occurring in over 50% of cases [5] and *NRAS* mutations, such as Q61R, observed in 15−20% of cases [6]. These mutations activate the MAPK (mitogen-activated protein kinase)/ERK (extracellular signal-regulated kinase signaling pathway (RAS, RAF, MEK1/2 (mitogen-activated extracellular signal-regulated kinase), and ERK1/2), promoting cell growth, proliferation, and migration, thus contributing to melanoma development and progression [5]. Targeted therapies, including FDA (Food and Drug Administration)-approved BRAF-inhibitors vemurafenib and dabrafenib, have significantly improved survival rates in BRAF^V600E^ melanoma patients [7,8]. However, the rapid emergence of resistance mechanisms, such as secondary *NRAS* mutations [9,10], highlights the need for combinatorial therapies, such as adding MEK inhibitors [11,12].

Vemurafenib is a targeted therapy that selectively blocks the MAPK/ERK signaling pathway in tumor cells carrying BRAF^V600E^ mutation [5,13,14]. Despite this effect in *BRAF* mutated melanoma [15], it conversely increases ERK signaling in melanoma cells exhibiting *RAS* mutation or wild-type *BRAF* [9,16,17,18,19]. In this regard, we recently showed that vemurafenib disrupts mitochondrial dynamics in BRAF^V600E^-mutated melanoma cells through the inhibition of the MAPK/ERK pathway, decreasing DRP1 activation and increasing MFN1/2 (mitofusin 1/2) and OPA1 (optic atrophy 1) levels, which results in a hyperfused mitochondrial phenotype [20]. However, the effects of vemurafenib in *NRAS*-mutated melanomas have not yet been described.

RAS (rat sarcoma) activation, in response to an extracellular signal through the binding to GTP (guanosine triphosphate), activates downstream signaling, after which GTP is hydrolyzed to GDP (guanosine diphosphate) and the RAS protein becomes inactive again [21]. *NRAS* mutations permanently lock the protein in a signal-emitting mode, even in the absence of growth factors, so they are constitutively active with a sustained cell proliferation signal [21,22]. Clinical trials with MEK inhibitors as monotherapy have not been shown to be effective in this type of cancer [23,24] or as an alternative to the use individualized use of these inhibitors, although some combination therapy studies are still ongoing [25,26,27]. Despite the lack of specific inhibitors for oncogenic *RAS*, it seems that RAS blockade by lonafarnib, preventing its translocation to plasma membrane, increase the efficacy of other chemotherapeutic agents and can be used in a combined regimen [28]. Considering that *NRAS* mutations are frequently reported as resistance mechanisms in *BRAF*-mutated melanomas treated with BRAF inhibitors [9,10,29], we investigated the effects of vemurafenib on melanoma cells harboring the NRAS^Q61R^ mutation. Specifically, we focused on alterations in mitochondrial energetics and dynamics to enhance our understanding of vemurafenib resistance mechanisms and to identify potential new therapeutic targets for combination therapy.

## 2. Results

### 2.1. Vemurafenib Has Low Cytotoxicity in NRAS^Q61R^-Mutated SK-MEL-147 Melanoma Cells and Conversely Induces Its Proliferation

**Figure 1:**
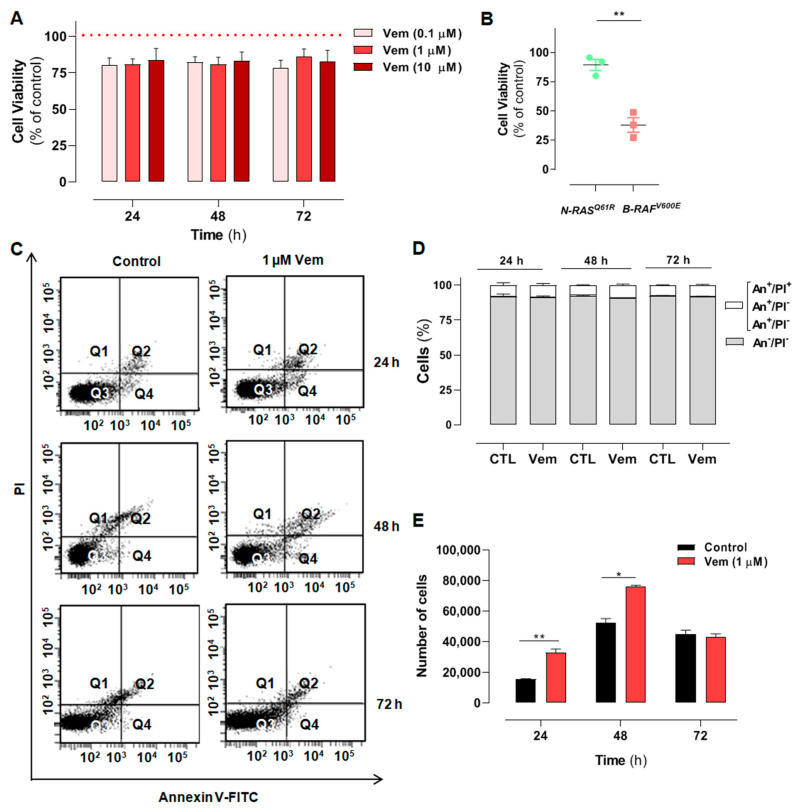

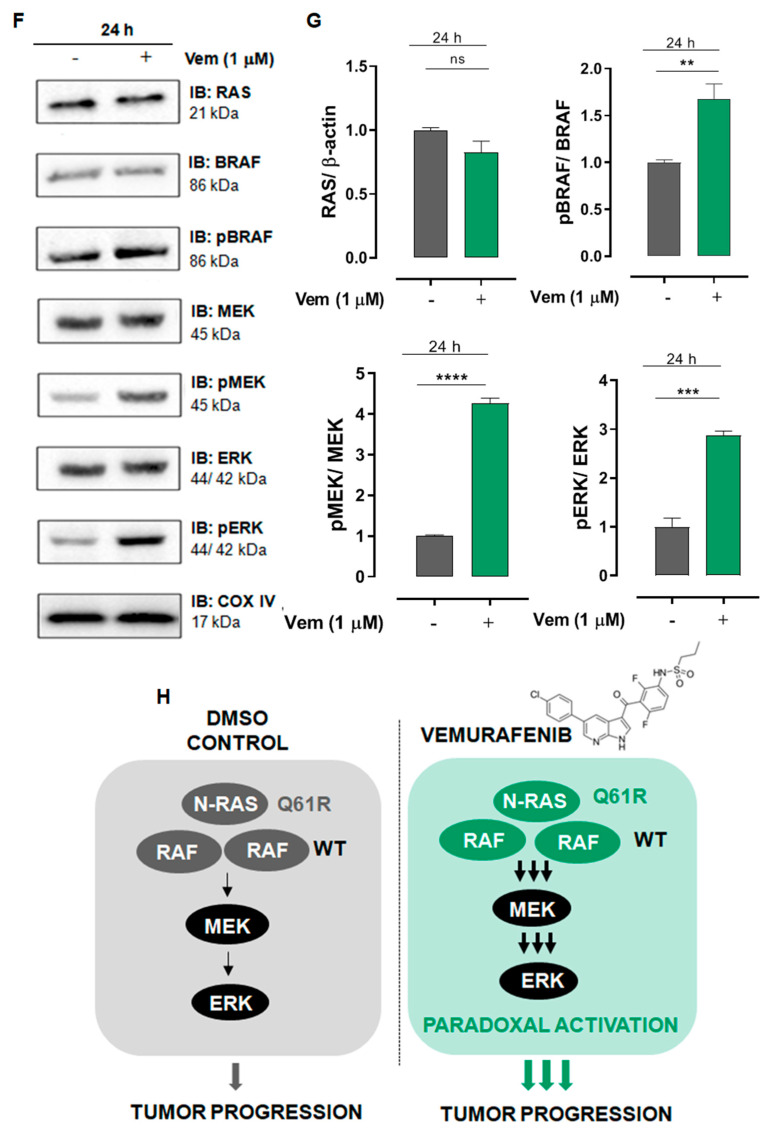
Melanoma cells presenting *NRAS* mutation are not sensitive to the cytotoxicity of vemurafenib and display increased proliferation rate associated with the paradoxical activation of the MAPK/ERK pathway. (**A**) SK-MEL-147 cell was treated with vemurafenib (0.1, 1 or 10 µM) or DMSO (control) for 24, 48, and 72 h, and cytotoxicity was estimated by the MTT reduction assay. The dotted red line represents the control, considered as 100%. The results are presented as mean ± SEM (standard error of mean) of three independent experiments performed in triplicate. (**B**) Scatter plot comparing the cytotoxic effect of 1 µM vemurafenib incubated for 72 h with SK-MEL-147 (*NRAS* mutant) or SK-MEL-19 (*BRAF* mutant) assessed by MTT. The results are presented as the mean ± SEM of three independent experiments performed in triplicate. Statistical significance of the data was performed using the unpaired *t*-test variance, considering ** *p* < 0.01. Flow cytometry analysis of SK-MEL-147 cells labeled with annexin-V FITC and propidium iodide (PI). (**C**) Representative dot plot of annexin-V FITC versus propidium iodide (PI) fluorescence intensity after cell treatment with 1 µM vemurafenib at 24, 48 and 72 h. (**D**) Quantification of annexin-V FITC and/or propidium iodide positive cells, presented as mean ± SEM of two independent experiments. (**E**) Growth curve of SK-MEL-147 after cell treatment with 1 µM vemurafenib (red bars) at 24, 48 and 72 h. The results are presented as the mean ± SEM of two independent experiments performed in triplicate. Statistical significance of the data was performed using the unpaired *t*-test variance, considering * *p* < 0.1 and ** *p* < 0.01. SK-MEL-147 total protein lysates were obtained after incubation with 1 µM vemurafenib or DMSO (control) for 24 h. (**F**) Representative Western blotting bands showing the expression of BRAF, pBRAF, MEK, pMEK, ERK, pERK, and COX IV or β-actin (internal control). Aliquots of 50 and 100 μg of protein were used in each well for total proteins and their phosphorylated forms, respectively. (**G**) The quantification of bands was performed by densitometry, followed by normalization using β-actin. The phosphorylated forms were also normalized by their respective total pair (ratio phosphorylated/total) and the mean of control (without vemurafenib) was normalized to 1. The results are presented as mean ± SEM of three independent experiments. Statistical significance of data was performed using the unpaired *t* test variance, considering ns = not significant, ** *p* < 0.01, *** *p* < 0.001, and **** *p* < 0.0001. (**H**) Representative illustration of the MAPK pathway in melanoma cells with the NRAS^Q61R^ mutation. On the left, basal signaling, and on the right, paradoxical activation, caused by vemurafenib action.

The human NRAS^Q61R^ mutant SK-MEL-147 melanoma cells were incubated with increasing concentrations of vemurafenib (0.1, 1, and 10 μM) for 24, 48, or 72 h, and the percentage of viable cells was calculated in relation to control (same volume of vehicle DMSO, dimethyl sulfoxide) and evaluated by the MTT (3-(4,5-dimethyl-2-thiazolyl)-2,5-diphenyl-2H-tetrazolium bromide) reduction assay. Time- and concentration–response curves showed that viability was not significantly affected by vemurafenib, regardless of concentration and incubation time (Figure 1A). This is in agreement with the literature, as vemurafenib does not exert cytotoxicity in melanoma cells presenting the *NRAS* mutation. Furthermore, to validate the targeted vemurafenib effect and the experimental model, we compared its effect with those obtained in SK-MEL-19 cells (BRAF^V600E^ mutant), which are sensitive to vemurafenib, as previously described by our group [20]. Thus, as expected, 1 µM vemurafenib decreased the viability of mutant *BRAF* cells by 62% after 72 h of incubation (Figure 1B, red squares). Under the same conditions, *NRAS* mutant cells were poorly responsive to vemurafenib, which decreased cell viability only by 14% (Figure 1B, green dots). This effect on viability was also investigated by flow cytometry using the double-labeling assay with annexin V (An)-FITC (fluorescein isothiocyanate) and propidium iodide (PI) to evaluate phosphatidylserine externalization during an apoptotic process or plasma membrane permeabilization, as occurs in necrosis [30]. After cell incubation for 24, 48, or 72 h with 1 µM vemurafenib, the fluorescence emission profiles of FITC and PI were similar to the control at all different times. A large percentage of viable cells (Q_4_/An^−^PI^−^) and a low percentage of cells in early or late apoptosis/secondary necrosis (Q_3_/An^+^PI^−^, Q_2_/An^+^PI^+^, and Q_1_/An^−^PI^+^) were observed in the representative dot plots (Figure 1C,D). This result provides additional evidence for the low cytotoxicity of vemurafenib in NRAS^Q61R^ mutant melanoma cells. Moreover, to further evaluate the possible effect of vemurafenib on cell proliferation, a growth curve of SK-MEL-147 cells treated with 1 µM vemurafenib was also carried out, monitored for 24, 48, and 72 h (Figure 1E, red bars), and an increase in cell proliferation was observed in 24 and 48 h in relation to control (black bars).

### 2.2. Vemurafenib Induces Paradoxical Activation of the MAPK/ERK Pathway in NRAS^Q61R^-Mutated Melanoma Cells

The mechanism of action of vemurafenib consists of inhibiting the mutant BRAF^V600E^ protein monomers and consequently blocks all downstream signaling targets in the MAPK/ERK pathway. However, in melanoma cells presenting the *NRAS* mutation, vemurafenib is thought to inhibit only one RAF promoter in the dimer, whereas the other one remains active and maintains signals for downstream activation of MAPK/ERK pathway. To test this, we analyzed the effects of vemurafenib on the expression of all proteins of the cascade, i.e., RAS, BRAF, pBRAF, MEK, pMEK, ERK, and pERK, in SK-MEL-147 cells after 24 h of incubation. The total amount of the proteins was not altered by vemurafenib. However, instead of inhibiting MAPK/ERK cascade, as observed in BRAF^V600E^ melanoma cells, vemurafenib significantly increased the phosphorylation of RAF, MEK, and ERK (Figure 1F,G), promoting the paradoxical activation of the MAPK/ERK pathway in the NRAS^Q61R^ cell line, as depicted in Figure 1H. Such overactivation of the MAPK/ERK signaling by vemurafenib in SK-MEL-147 cells can explain its increased proliferative rate observed in Figure 1E. Cancer homeostasis depends on a balance between activated oncogenic pathways that drive tumorigenesis and the commitment of the activation of stress response programs that counteract the inherent toxicity of this aberrant signaling. Although inhibition of oncogenic signaling pathways has been widely explored, there is increasing evidence showing that hyperactivation of these same pathways can also disrupt cancer homeostasis and cause lethality [31,32,33,34]. This scenario suggests that the deliberate hyperactivation of oncogenic signaling pathways, as the MAPK pathway, in cancer cells can lead to an extreme dependence on stress response pathways, creating possible vulnerabilities [35].

### 2.3. Vemurafenib Does Not Cause Extensive Mitochondrial Fusion in NRAS-Mutated Melanoma Cells

**Figure 2:**
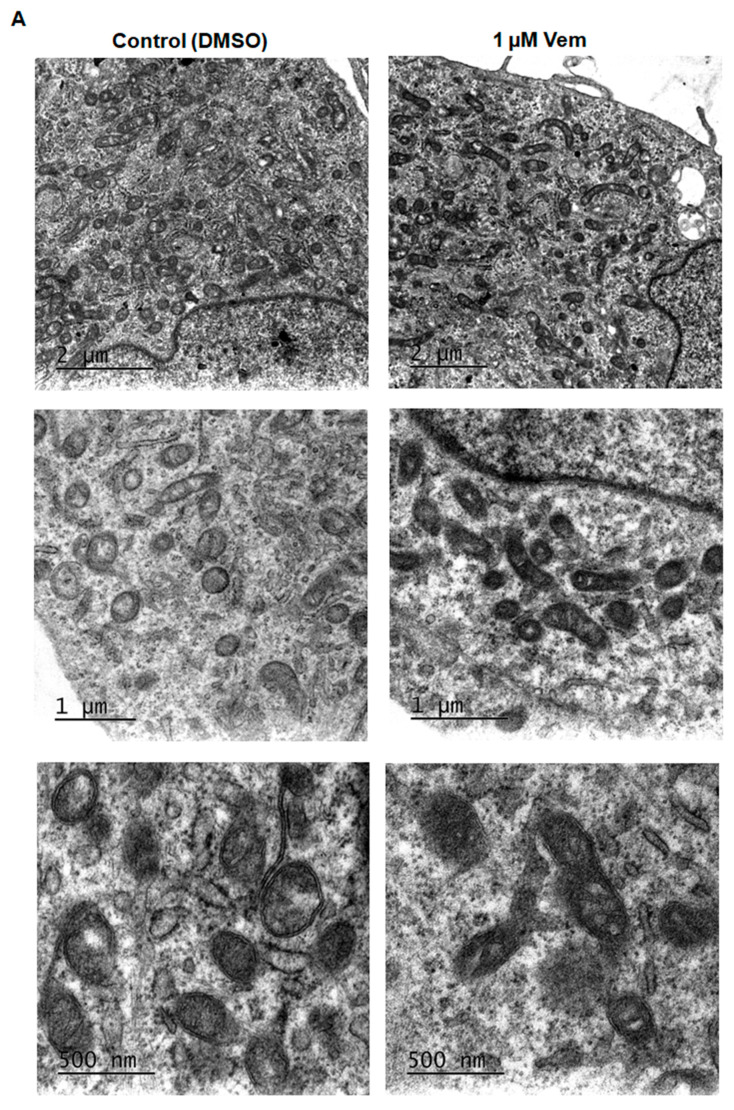

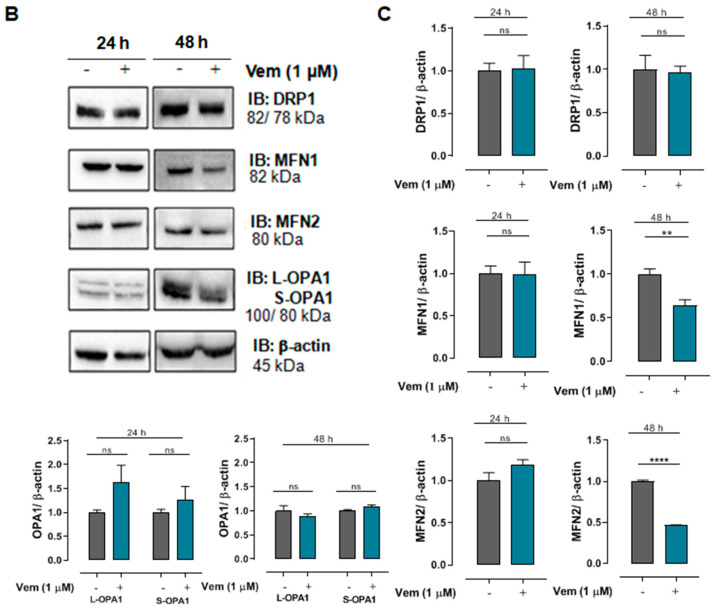
Vemurafenib does not induce hyperfused mitochondrial morphology in NRAS^Q61R^ mutant melanoma cells. (**A**) Representative photomicrograph of mitochondrial morphology in SK-MEL-147 cells obtained by transmission electron microscopy after cell incubation with 1 µM vemurafenib or with DMSO (control) for 24 h. The scale bars represent 2 µm (upper panels), 1 µm (middle panels), and 500 nm (lower panels). (**B**) Cell lysates were obtained after incubation of SK-MEL-147 cells with 1 µM vemurafenib or DMSO (control) for 24 and 48 h. The panels show the Western blotting bands of DRP1, MFN1, MFN2, L-OPA1, S-OPA1, and β-actin (load control). Aliquots of 50 µg of protein were used in each lane for total proteins and their phosphorylated forms, respectively. (**C**) Quantification of bands was performed by densitometry, followed by normalization with the corresponding β-actin. The results obtained are presented as the mean ± SEM of three independent experiments. The mean of the controls was normalized to 1 and that of the treated was calculated according to the control. Statistical significance of data was performed using the unpaired *t* test variance, considering ns = not significant, ** *p* < 0.01, and **** *p* < 0.0001.

The inhibition of MAPK/ERK cascade by vemurafenib results in extensive mitochondrial fusion in BRAF^V600E^ melanoma cells [20]. Considering that MAPK/ERK cascade regulates mitochondrial fission and fusion processes, mitochondrial morphology was first analyzed in SK-MEL-147 cells treated with vemurafenib 1 μM or DMSO (control) for 24 h by transmission electron microscopy. Differently from the hyperfused phenotype observed in melanoma cells with a *BRAF* mutation [20], SK-MEL-147 cells incubated with vemurafenib exhibited smaller, rounded mitochondria, similar to the control, suggesting mitochondrial fission prevalence (Figure 2A). To assess potential alterations in the expression of proteins involved in the balance between mitochondrial fission and fusion, total protein lysates were prepared after incubation of cells with 1 µM vemurafenib or DMSO (control) for 24 or 48 h, followed by Western blotting. The phosphorylated form of DRP1 was not detected by this technique. After 24 h of incubation, no statistically significant differences were observed in the expression of DRP1, MFN1, MFN2, and OPA1. However, after 48 h of incubation, a significant decrease in MFN1 and MFN2 expression was observed (Figure 2B,C). These changes may be attributed to the paradoxical activation of the MAPK pathway by vemurafenib in NRAS^Q61R^ melanoma cells.

### 2.4. Combined Treatment with Mdivi-1 Synergistically Sensitizes NRAS^Q61R^ Melanoma Cells to Vemurafenib

**Figure 3:**
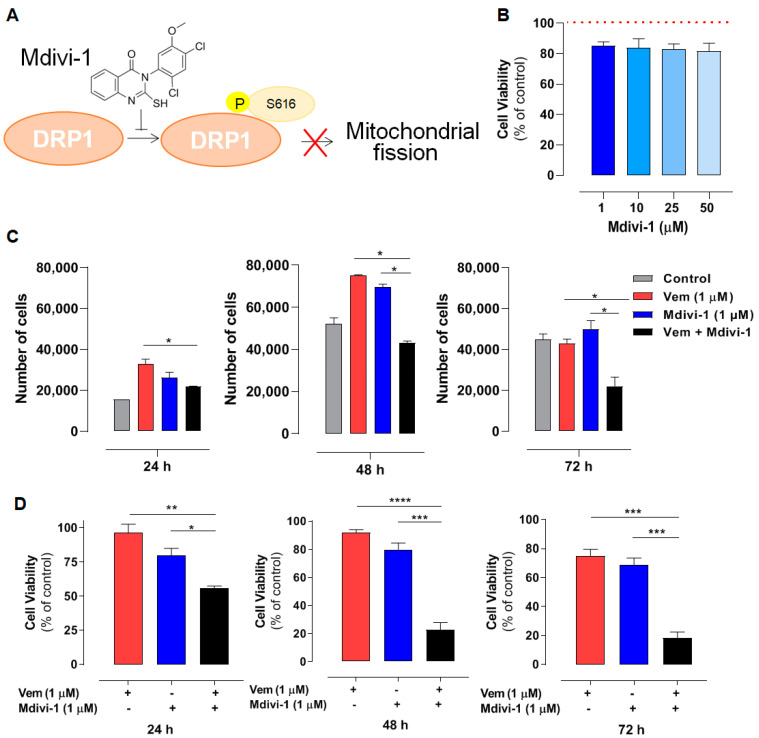

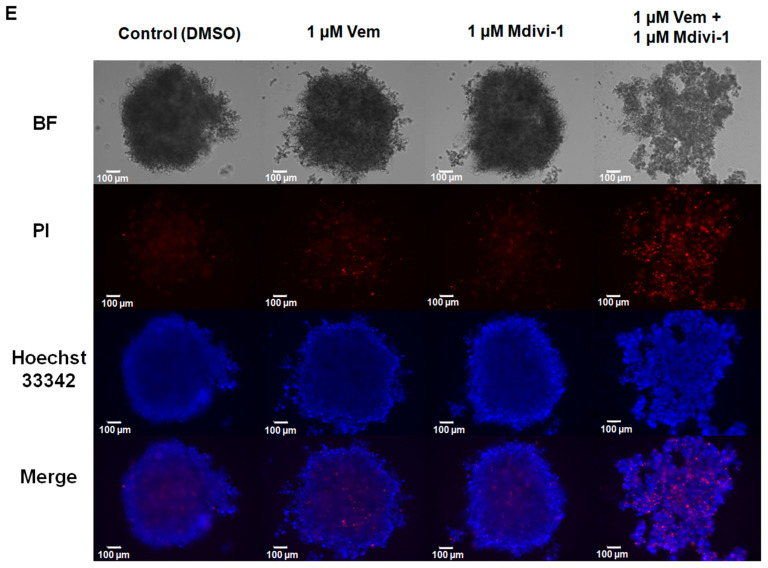
Mdivi-1 sensitizes NRAS^Q61R^ melanoma cells to vemurafenib. SK-MEL-147 cells were pre-incubated with 1 µM mdivi-1 for 1 h and then incubated with 1 μM vemurafenib or with DMSO (control) for 24, 48, and 72 h. (**A**) Representative scheme illustrating the mechanism of action of mdivi-1, targeting the DRP1 protein, preventing its phosphorylation at serine 616. (**B**) Evaluation of the effect of mdivi-1 (1; 10; 25, and 50 µM) on cell viability after 24 h of incubation. Dotted red line represents the control, considered as 100%. The results are presented as mean ± SEM of three independent experiments. (**C**) Cells (188,000 per well) were incubated with vemurafenib (red bar), mdivi-1 (blue bar) or vemurafenib + mdivi-1 (black bar) combination (at 1 µM each one) for 24, 48 or 72 h, followed by counting in a Neubauer chamber using the trypan blue 0.016% (*w*/*v*). Data are presented as mean ± SEM of two independent experiments. Statistical significance of data was performed using ANOVA (one-way analysis of variance) and Tukey’s post-test, * *p* < 0.05. (**D**) Evaluation of the cytotoxicity of vemurafenib (red bar), mdivi-1 (blue bar) or their combination (black bar) by MTT. Data are presented as mean ± SEM of three independent experiments. Statistical significance of data was performed using ANOVA and Tukey’s post-test, * *p* < 0.05, ** *p* < 0.01, *** *p* < 0.001 and **** *p* < 0.0001. (**E**) The spheroids (6000 cells and 72 h of formation) were treated with vemurafenib, mdivi-1 or vemurafenib + mdivi-1 combination (at 1 µM each one) for 24 h, then stained with 1.25 µM propidium iodide and 4.0 µM Hoechst 33342 for 30 min. The images were acquired using fluorescence microscopy at 100× magnification. The scale bars represent 100 µm.

Since vemurafenib altered the expression of mitochondrial dynamic proteins, we further explored the contribution of DRP1 in the maintenance of mitochondrial morphology observed in NRAS^Q61R^ melanoma. To do this, mdivi-1was used, which is a selective inhibitor of DRP1 phosphorylation and, consequently, an inhibitor of mitochondrial fission (Figure 3A) [36]. Firs, a concentration–response curve was generated to assess the effects of mdivi-1 on SK-MEL-147 cell viability; mdivi-1 showed no significant cytotoxicity after 24 h of incubation at concentrations up to 50 µM (Figure 3B). Although vemurafenib or mdivi-1 did not exhibit significant cytotoxicity in SK-MEL-147 cells, their effects on cell growth have not yet been investigated. Thus, to assess the possible effect of vemurafenib, mdivi-1, or their combination on cell proliferation, a Neubauer chamber count was performed after 24, 48, and 72 h of incubation. It is important to point out that the same number of cells was platted in each well to start the experiment. As expected, the control growth (absence of drugs) showed a time-dependent growth with the maximum number of cells at 48 h (Figure 3C, gray bar). Notably, vemurafenib significantly increased the number of cells after 24 h and 48 h of incubation compared to the control (Figure 3C, red bar), probably due to the paradoxical activation. This effect was also observed with 1 μM mdivi-1 (Figure 3C, blue bar). Interestingly, the combination of vemurafenib and mdivi-1 significantly decreased SK-MEL-147 cell proliferation after 24, 48, and 72 h of incubation (Figure 3C, black bar). These results suggest that the inhibition of mitochondrial fission in melanoma cells with the NRAS^Q61R^ mutation induces a notable antiproliferative effect, indicating that it may represent a potential therapeutic target for this type of cancer. Thus, we further evaluated the effects of this combination on cell viability by MTT. Consistently with Figure 1 and Figure 2, neither vemurafenib (Figure 3D red bar) nor mdivi-1 (Figure 3D, blue bar) alone exerted significant cytotoxicity on *NRAS*-mutant cells, regardless of the incubation time. However, their combination significantly reduced cell viability at all incubation times, with a more pronounced effect observed after 48 and 72 h (Figure 3D, black bars). Further calculations of the combination index (CI) revealed a synergistic effect between vemurafenib and mdivi-1 (Table 1). CI values less than one indicate a synergistic interaction: the closer to zero, the greater the synergistic effect [37].

Thereafter, we also evaluated the effect of combining vemurafenib with mdivi-1 after 24 h of incubation in spheroids generated from the SK-MEL-147 cell line. Tridimensional tumor cell cultures introduce complexity, providing nutrients and oxygen gradients similar to those experienced by solid tumors in patients. Spheroids incubated with 1 μM vemurafenib, mdivi-1, or their combination, and stained with Hoechst 33342 for live and PI for dead cells. Vemurafenib or mdivi-1 alone did not interfere with spheroid formation or increased cell death (red staining). However, the combination of both drugs increased cell death and disrupted the spheroid structure. All these findings highlight that mdivi-1-mediated inhibition of mitochondrial fission sensitizes *NRAS*-mutant melanoma cells to the antitumor effects of vemurafenib.

### 2.5. Mdivi-1 Reverts the Paradoxical Activation of MAPK/ERK Pathway Elicited by Vemurafenib in SK-MEL-147 Cells

**Figure 4:**
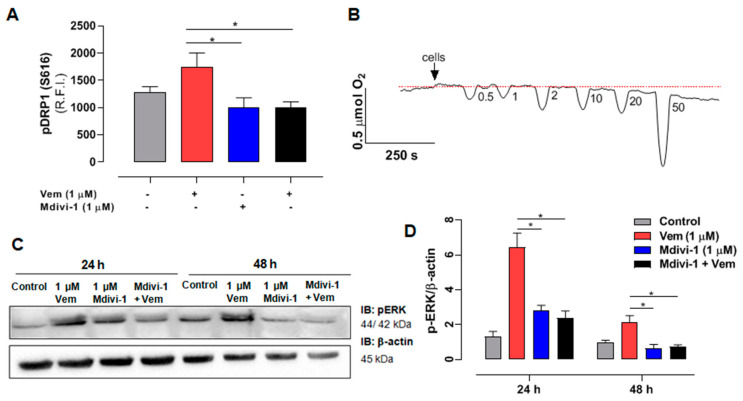
Mdivi-1 blocks the paradoxical activation of the MAPK/ERK cascade induced by vemurafenib in NRAS^Q61R^ melanoma cells. (**A**) Quantification of relative fluorescence intensity (R.F.I.) of pDRP1 (S616) incubation with vemurafenib (red bar) and mdivi-1 (blue bar) or both (black bar) (at 1 μM) or DMSO (control, gray bar) for 24 h. The data are presented as mean ± SEM of three independent experiments. Statistical significance of data was performed using ANOVA and Tukey’s post-test, * *p* < 0.05. (**B**) Oxygen consumption rate in the SK-MEL-147 cells. After the addition of cells indicated by the arrow, the basal consumption rate was established (pointed by the red dotted line), and the effects of sequential additions of mdivi-1 resulting in increasing concentrations (0.5; 1; 2; 10; 20; and 50 μM) was measured. (**C**) SK-MEL-147 total cell lysates were obtained after incubation with vemurafenib and/or mdivi-1 (at 1 μM) or DMSO (control) for 24 h. Representative immunoblotting pERK in melanoma cells with the NRAS^Q61R^ mutation. (**D**) Quantification of bands was performed by densitometry, followed by normalization with the corresponding β-actin, vemurafenib (red bar), and mdivi-1 (blue bar) or both (black bar) (at 1 μM) and DMSO (control—gray bar) for 24 h. The data are presented as mean ± SEM of two independent experiments. Statistical significance of data was performed using ANOVA and Tukey’s post-test, * *p* < 0.05.

Considering that mdivi-1 sensitized *NRAS*-mutant melanoma cells to the action of vemurafenib, and that DRP1 is a downstream target for phosphorylation of active ERK [38], we investigated whether this effect was related to its inhibition of DRP1 phosphorylation and consequent inhibition of mitochondrial fission. As expected, considering the paradoxical activation of MAPK/ERK pathway, vemurafenib increased DRP1 phosphorylation/activation (Figure 4A, red bar), and this effect was completely abolished by mdivi-1 (Figure 4A, black bar). Furthermore, since recent studies suggest that mdivi-1 can inhibit complex I of the mitochondrial respiratory chain [39,40], we aimed to distinguish whether the effects were not due to DRP1 inhibition, but rather from the respiratory chain inhibition, by evaluating the effect of increasing concentrations of mdivi-1 on O_2_ consumption in SK-MEL-147 cells. The representative trace in Figure 4B shows that mdivi-1 did not affect the O_2_ consumption rate at 0.5, 1, or 2 μM compared to the basal levels. However, at 10 μΜ, a slight decrease in O_2_ consumption was observed, which became more pronounced at 20 and 50 μM. Therefore, mdivi-1 sensitized SK-MEL-147 cells to vemurafenib in a concentration that does not inhibit the mitochondrial respiratory chain, suggesting that such effect is due to the inhibition of mitochondrial fission. We also investigated the possibility of mdivi-1 interferes with ERK phosphorylation, with or without vemurafenib (Figure 4C,D).

As already shown, vemurafenib (Figure 4D, red bar) increased pERK levels (paradoxical activation), but, surprisingly, the combined treatment of mdivi-1 with vemurafenib (Figure 4C, black bar) blocked the paradoxical activation, bringing pERK expression to levels similar to those of the control. This suggests that, beyond its direct inhibitory effect on DRP1 phosphorylation, mdivi-1 might target an upstream component of the MAPK pathway, which remains to be identified.

### 2.6. Interference with Mitochondrial Bioenergetics Also Sensitizes NRAS^Q61R^ Melanoma Cells to Vemurafenib

**Figure 5:**
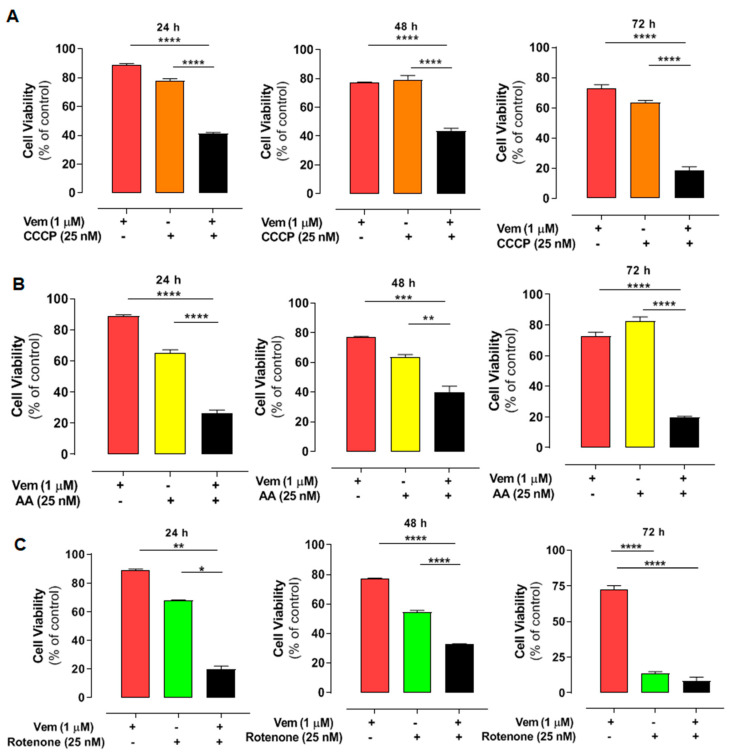
Combined effect of vemurafenib and mitochondrial interferents on the viability in NRAS-mutated melanoma cells. The cells were pre-incubated with CCCP (orange bars) (**A**), antimycin A (AA—yellow bars), (**B**) or rotenone (green bars) (**C**), all at 25 nM concentration for 1 h. After that, 1 μM vemurafenib or DMSO (control) was added followed by incubation for 24, 48, and 72 h. Red bars are the effects of vemurafenib only and black bars, the combination of vemurafenib with each modulator. The percentage of viable cells was calculated in relation to the control (absence of drug), and the data are presented as mean ± SEM of three independent experiments. Statistical significance of data was performed using ANOVA and Tukey’s post-test, considering * *p* < 0.05, ** *p* < 0.01, *** *p* < 0.001, and **** *p* < 0.0001.

Considering the effects of mdivi-1 on mitochondria, we also evaluated the impact of the disruption of mitochondrial bioenergetics in NRAS^Q61R^ cells treated with vemurafenib. We used CCCP as an uncoupler of oxidative phosphorylation (Figure 5A, orange bars), antimycin A (AA) as an inhibitor of respiratory complex III (Figure 5B, yellow bars), and rotenone as an inhibitor of complex I (5C, green bars). After doing concentration–response curves, we selected low cytotoxic concentrations for all modulators. Thus, cell viability was assessed using 1 µM vemurafenib and 25 nM of each modulator and their combinations after 24, 48, or 72 h of incubation (Figure 5). Vemurafenib or the modulators alone did not exhibit high cytotoxicity. However, their combinations significantly exacerbated the cytotoxicity of vemurafenib in *NRAS*-mutated melanoma cells. The CI analyses revealed a synergistic effect for most combinations after 24, 48, and 72 h (Table 2). Thus, interfering with mitochondrial bioenergetics may also sensitize NRAS^Q61R^ melanoma cells to vemurafenib, highlighting mitochondria as a potential therapeutic target in this type of cancer.

### 2.7. The Expression of Genes Involved in Mitochondrial Dynamics Is Altered in NRAS-Mutated Melanoma Samples from Patients

**Figure 6:**
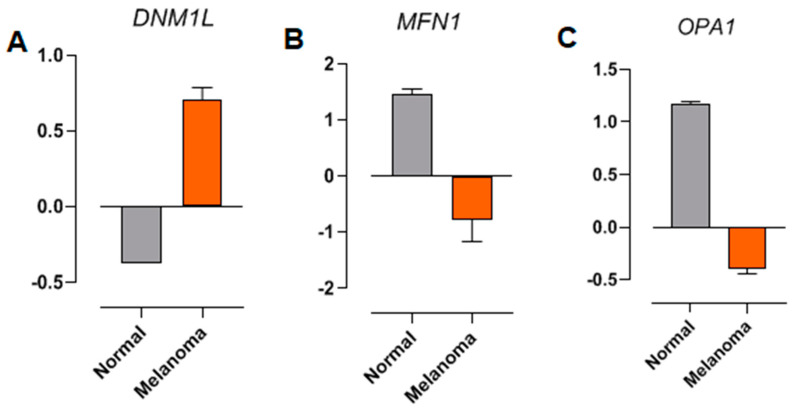
Differential expression analysis of (**A**) *DNM1L* (DRP1), (**B**) *MFN1*, and (**C**) *OPA1*. RNA sequencing from patient sample databases. The raw RNA sequencing data from normal samples (gray bars) and melanoma (orange bars) associated with NRAS-type mutations were obtained from a previous study [36].

The raw RNA sequencing data of *DNML1*, *MFN1*, and *OPA1* from normal and melanoma samples, associated with *NRAS*-type mutations, were analyzed from a previous study [41]. Differential expression (DE) analysis was performed to compare the transcriptomic profiles of melanoma samples with those of normal tissues (control). The expression of *DNML1* (encoding the human *DRP1* gene, a key regulator of mitochondrial fission) is increased in *NRAS*-mutated melanoma samples compared to the normal tissues (Figure 6A). In contrast, transcripts encoding proteins critical for mitochondrial fusion and cristae organization, such as *MFN1* and *OPA1*, were downregulated at the same samples (Figure 6B and Figure 6C, respectively). These findings corroborate the in vitro results presented here and indicate the impact of *NRAS* mutations on mitochondrial dynamics.

## 3. Discussion

Author-targeted therapy with vemurafenib allowed significant advances in the treatment of *BRAF*-mutated metastatic melanoma. However, *NRAS*-mutated melanomas still lack a specific inhibitor or chemotherapeutic drug for their treatment, remaining neglected. The strategy of inhibiting NRAS downstream proteins in the MAPK/ERK pathway is not effective. Moreover, BRAF inhibitors (vemurafenib, dabrafenib, and encorafenib) cannot be used as single drugs to treat mutant *NRAS* melanoma, because they paradoxically increase the MAPK/ERK signaling and, consequently, tumor proliferation [42,43].

Despite the therapeutic success of RAF and MEK inhibitors in the treatment of BRAFV600-mutated tumors, one major remaining challenge is the unavoidable emergence of resistance to these inhibitors, frequently by up-regulation of the MAPK pathway through *NRAS*, *KRAS*, or *MEK* mutations, and also by *BRAF* amplification or alternative splicing [44]. It is noteworthy that these resistant tumors are generally sensitive to drug withdrawal, indicating that hyperactivation of the MAPK pathway is not tolerated [33]. This strongly suggests that alterations in oncogenic signaling levels can disrupt the fragile homeostasis of cancer cells, with up-regulation or oncogene overdose being potentially as harmful as the down-regulation or inhibition of the pathway, pushing these cells to the limit of viability [31,34]. In this sense, additional activation of mitogenic pathways should increase the dependence of cancer cells on stress response pathways [34]. Overexpression of ERK2 due to hyperactivation of MAPK signaling can sensitize only *RAS*/*RAF* mutant melanoma, but not *RAS*/*RAF* wild-type melanoma, suggesting that cells must maintain tightly regulated MAPK signaling levels, which can promote tumor growth but are not tolerated above a given threshold [33].

Drug-naive *NRAS*-mutant cells are sensitive to the silencing of DUSP4 (dual specificity phosphatase 4), a protein tyrosine phosphatase that selectively dephosphorylate and inactivate ERK. The depletion of DUSP4 induce oncogene overdose in both drug-naïve and drug-resistant *BRAF*-mutant melanoma cell lines [32,45] through the hyperactivation of MAPK signaling. Resistant cells to MAPK inhibitors become sensitive after disruption of DUSP4 and DUSP6 (dual specificity phosphatase 6) [46]. Also, the hyperactivation of MAPK signaling increases reactive oxygen species (ROS) levels in BRAF inhibitor-resistant melanoma cells [34], and histone deacetylase inhibitor vorinostat boosts ROS levels, selectively killing drug-resistant tumor cells [47]. Accordingly, it has been recently shown that *NRAS*-mutated melanomas have lower PRDX2 (peroxiredoxin 2) expression compared to *BRAF*-mutated melanomas, which is correlated with lower survival and higher malignancy in patients. The antioxidant gliotoxin is able to decrease migration and invasiveness of those cells [48]. Interestingly, PRDX2 can interfere with the cellular oxidative metabolism, and it is depleted in vemurafenib-resistant melanomas [49]. In vitro studies have also provided encouraging perspectives: for example, the combination of vemurafenib and binimetinib amplifies pro-apoptotic activity in melanoma with *NRAS* mutation [25]. It has been proposed that NRAS^Q61R^ mutants seem to depend particularly on glucose metabolism and that metabolic stress can sensitize this mutant to the action of sorafenib [50]. We have shown that, despite the absence of cytotoxicity, vemurafenib increases MAPK/ERK signaling by paradoxical activation in NRAS^Q61R^ melanoma, which may be associated with increased tumor proliferation.

In fact, mitochondrial metabolism has emerged as a very promising therapeutic target in cancer treatment [51], and, in this scenario, changes in mitochondrial dynamics have gained attention. Many studies point out that tumor cells sustain the mitochondrial fission phenotype [52,53,54,55,56]. Such a feature would be related to quality control, removing damaged mitochondria and facilitating apoptosis in cells under high levels of stress [57]. In a previous study, it was shown that vemurafenib induces extensive mitochondrial fusion in BRAF^V600E^ melanoma [20]. Apparently, this phenotype with more elongated mitochondria is associated with increased oxidative phosphorylation (OXPHOS) capacity. Conversely, in NRAS^Q61R^ melanoma, a smaller and rounded morphological mitochondrial profile was observed, indicating mitochondrial fission. Compared to *BRAF* mutants, it was verified that *NRAS* mutants have higher pDRP1 expression and a decrease in fusion proteins (MFN1 and MFN2). Thus, DRP1 is considered a pivotal regulator of oncogenic transformation and impacts the outcome of chemotherapy [58].

Given the importance of mitochondrial dynamics in tumor metabolism and tumorigenesis, its modulation can represent a relevant therapeutic approach. Understanding the molecular mechanisms underlying mitochondrial dynamics enabled the development of several compounds that promote its modulation [59]. Enhancement of fusion through MFN2 expression may control cell proliferation [60,61,62]. In contrast, inhibition of fission decreases proliferation and increases apoptosis in lung cancer [55], colon cancer [54], breast cancer [56], and thyroid cancer [63]. Here, we modulated mitochondrial dynamics of *NRAS* melanoma using mdivi-1, a DRP1 inhibitor, which sensitized *NRAS* mutant melanoma to vemurafenib. Moreover, the combined treatment was able to reverse the paradoxical activation of the MAPK proteins, reaching levels of pERK expression similar to the basal, suggesting that mdivi-1 might have an unknown role in the regulation of this pathway. It has already been reported that this strategy of combined therapy with mdivi-1 can increase the cytotoxicity of other drugs. For example, mdivi-1 enhanced the antitumor effect of taxol in highly refractory triple negative breast cancer [64] in combination with cisplatin in renal carcinoma cells [65] and ovarian cancer [66] with venetoclax in acute myeloid leukemia cells [67], both with gemcitabine in pancreatic ductal adenocarcinoma [68] and with platinum drugs in hepatocellular carcinoma by modulating mitochondrial dynamics [69]. The possible antiproliferative activity of mdivi-1 is poorly described, except for gastric [70] and thyroid tumor cells [63]. Thus, its antiproliferative effect in melanoma cells presenting *NRAS* mutation is unprecedented, and, moreover, it may be of great therapeutic relevance.

It has been proposed that mdivi-1 displays other nonspecific effects (off target) besides DRP1 inhibition, including the reversible inhibition of mitochondrial complex I, which was achieved only at 50 to 100 μM in COS-7 cells and primary cortical neurons [39]. It is expected that, at very high concentrations, other non-specific effects of mdivi-1 can be found. In *C. albicans*, 30 μM mdivi-1 decreased hyphae growth by a mechanism involving metabolic reprogramming and a drastic reduction in endogenous nitric oxide levels [40]. We believe that mdivi-1 (at 1 μM) sensitized *NRAS* mutant melanoma cells to vemurafenib due to the inhibition of DRP1. However, other undisclosed effects can contribute to this effect. Metabolic reprogramming has become one of the hallmarks of cancer, and OXPHOS is active in many cancer models, including melanoma, even under hypoxic conditions [71]. Pharmacological strategies inhibiting OXPHOS and/or AMPK (AMP-activated protein kinase) and suppress cell proliferation in vitro and in vivo in melanoma have already been explored, for example, with the use of AMPK inhibitors AICAR (5-aminoimidazole-4-carboxamide-ribonucleoside) [72,73] and GSK621 [74]. Also, other OXPHOS inhibitors, such as biguanides (metformin and phenformin) [72,73,75,76] and BAY 87-2243, which target complex I of the respiratory chain [77], in addition to OXPHOS uncouplers, such as SR4 and niclosamide [78], have been studied. Our results corroborate these findings, showing that modulation of the respiratory chain can sensitize *NRAS* mutant melanoma cells to vemurafenib action. Further studies are required to assess these observations in other NRAS-mutant tumor cells in order to generalize the findings and also to explore these alterations in vemurafenib-resistant BRAF-mutant melanoma cells. Obviously, it is a proof of concept that interfering with mitochondrial dynamics and bioenergetics sensitizes *NRAS* mutant melanoma cells to vemurafenib, since these inhibitors and uncouplers have systemic toxicity and can compromise the energy homeostasis of other tissues, such as the heart and brain, making their therapeutic use unfeasible [78].

## 4. Materials and Methods

### 4.1. Three-Dimensional Cell Culture and Standard Incubation Conditions with Vemurafenib

Human melanoma SK-MEL-147 cells (NRAS^Q61R^ mutant/*BRAF* wild type) and SK-MEL-19 cells (BRAF^V600E^ mutant/*NRAS* wild type) were provided by Prof. Silvya Stuchi Maria-Engler (FCF-USP) in 2017. The cell lines were certified by short tandem repeat DNA profile and tested mycoplasma-free. The cells were grown in Dulbecco’s Modified Eagle’s medium (DMEM) high glucose (Sigma-Aldrich, St. Louis, MO, USA), pH 7.2, and supplemented with 10% fetal bovine serum (Invitrogen, Waltham, MA, USA), 100 U/mL penicillin, and 100 μg/mL streptomycin, in 5% CO_2_ at 37 °C. For assays, the cells were detached with trypsin/EDTA solution (Sigma-Aldrich, St. Louis, MO, USA), centrifuged (160× *g* for 10 min), and suspended in supplemented DMEM. Then, the cells (6.25 × 10^4^ cells/cm^2^) were pipetted into microplate wells and incubated during 24 h for cell adhesion. Vemurafenib (Selleck Chemicals, Houston, TX, USA) or other chemicals were added followed by additional incubation.

### 4.2. Three-DimensionalCell Culture (Spheroids) and Cell Viability Analysis

To produce the spheroids, SK-MEL-147 cells (6000 cells per well) were seeded in 96-well U-bottom plates coated with 2% agarose with supplemented DMEM high glucose. The plates were centrifuged at 160× *g* for 5 min and incubated for 72 h. The cells were treated with 1 μM vemurafenib and/or 1 μM mdivi-1 for 24 h. After incubation, the cells were stained with 1.25 µM propidium iodide (Sigma Aldrich, St. Louis, MO, USA, #P4170) and 4.0 µM Hoechst 33342 diluted in fluorescence buffer [20] for 30 min at 37 °C. Fluorescence emission was detected on a Leica AF6000 wide-field fluorescence microscopy (Leica Microsystems, Wetzlar, Germany).

### 4.3. Cytotoxicity Assays

Cytotoxicity of vemurafenib was evaluated by cell viability by MTT (Sigma-Aldrich, St. Luis, MO, USA) reduction assay, as previously described [79]. When applied, modulators were pre-incubated with cells for 1 h at the following concentrations: 25 nM CCCP (carbonyl cyanide 3-chlorophenylhydrazone; Sigma-Aldrich, USA), 25 nM AA (antimycin A; Sigma-Aldrich, USA), 25 nM rotenone (Sigma-Aldrich, USA), and 1 μM mdivi-1 (3-(2,4-dichloro-5-methoxyphenyl)-2,3-dihydro-2-thioxo-4(1H)-quinazolinone; Sigma-Aldrich, USA), followed by adding 1 μM vemurafenib (Selleck Chemicals, Houston, TX, USA). Cell viability was calculated in relation to the control (DMSO), which was considered 100%.

### 4.4. Growth Curves for Cell Proliferation

SK-MEL-147 cells (188,000 per well) were incubated for 24 h with 5% CO_2_ at 37 °C for cell adherence. Afterwards, the medium was replaced and 1 µM vemurafenib and/or 1 µM mdivi-1 was added, followed by incubation for 24, 48, or 72 h. The supernatant with detached cells by 1× trypsin was collected and, after inactivation by adding supplemented DMEM, the samples were centrifuged (270× *g* for 5 min) and the pellet resuspended in the culture medium. Dead and live cell counts were established using 0.016% (*w*/*v*) trypan blue in a Neubauer chamber.

### 4.5. Annexin V-FITC/PI Double Staining Assay

After 1 µM vemurafenib incubation for 24, 48, or 72 h, the SK-MEL-147 cells were detached, centrifuged (160× *g* for 10 min), and suspended in 50 µL of binding buffer [80] plus 5.0 µL Annexin V-FITC and 5.0 µL PI, according to the manufacturer’s recommendations (BD Biosciences, San Jose, CA, USA). The reaction mixture was incubated in the dark at 25 °C for 20 min. After dilution with an additional 0.3 mL of binding buffer, fluorescence emission was detected by a FACS Canto II Flow Cytometer (BD Biosciences, USA), acquiring 10,000 events per sample using a Coherent^®^ Sapphire™ 488-20 solid-state blue laser with an excitation at 488 nm, dichroic mirror 502 LP, band pass filter 530/30 for the FITC fluorescence, and dichroic mirror 556 LP, as well as a band pass filter 585/42 for the PI fluorescence. Data analysis and graphs were performed by using FlowJovX software vX.0.7 (Ashland, OR, USA).

### 4.6. High-Resolution Respirometry

Mitochondrial activity was measured using a high-resolution respirometer (Oxygraph-2k; Oroboros Instruments, Innsbruck, Austria) at 37 °C with magnetic stirring (750 rpm). The chemicals used to assess mitochondrial respiratory status were administered into separate chambers using Hamilton syringes (Hamilton Company, Reno, NV, USA), and the data were computed using DatLab 5.1.0.20 program (Oroboros Instruments GmbH, Innsbruck, Austria). Basal O_2_ consumption was established, and the effects of increasing concentrations of mdivi-1 (0.5, 1, 2, 10, 20, and 50 µM) on basal respiration was recorded.

### 4.7. Western Blotting

After incubations, the SK-MEL-147 cells were lysed in RIPA buffer 1× (Thermo Fisher Scientific, Waltham, MA, USA) containing 1× protease/phosphatase inhibitor cocktail (Cell Signaling, USA) and 200 nM PSMF (Cell Signaling Technology, Danvers, MA, USA). Protein concentration was determined by Lowry’s method. Briefly, protein mixture in cell lysates was resolved using sodium dodecyl sulfate–polyacrylamide gel electrophoresis (SDS–PAGE) and transferred to nitrocellulose membranes (Bio-Rad Laboratories, Hercules CA, USA). After membrane blocking with 2.5% BSA in TRIS-buffered saline containing Tween 20, proteins were subsequently detected using the following primary antibodies: β-actin (#3700), COX IV (#4844), MFN1 (#14739), MFN2 (#9482), OPA1 (#80471), DRP1 (#8570), RAS (#3965), BRAF (#14814), pBRAF(#2696), MEK 1/2 (#9126), pMEK 1/2 (#9154), p44/42 MAPK (ERK 1/2) (#4695), and p-p44/42 MAK (ERK 1/2) (#4695) (Cell Signaling, USA). After subsequent incubation with the respective HRP-conjugated secondary antibodies, anti-mouse (#7076) or anti-rabbit (#7074) (Cell Signaling, USA), the labeled proteins were revealed with chemiluminescent detection kit Pierce™ ECL Plus Substrate (Thermo Fisher Scientific, USA) and digitized using the ChemiDoc™ MP Imaging software v5.0 (Bio-Rad, USA).

### 4.8. Quantification of pDRP1 ser616

The SK-MEL-147 cells were seeded in black 96-well plates with a transparent bottom for cell adhesion for 24 h. After adding 1 µM vemurafenib and/or 1 µM mdivi-1, the samples were incubated for 24 h. The cells were fixed with PBS plus 2% paraformaldehyde for 30 min and the samples were washed twice with 300 µL of PBS plus 0.1 M glycine and permeabilized with PBS plus 0.01% saponin for 15 min. The primary antibody, pDRP1 (#4494, 1:500), was incubated overnight at 4 °C under orbital shaking. The secondary antibody (1:500), goat anti-rabbit IgG (#F8521), conjugated with Alexa Fluor 488, was incubated for 1 h at room temperature. The quantitative fluorescence measurement was performed in BioTek Synergy H1 Multi-Mode Reader (Agilent, Santa Clara, CA, USA).

### 4.9. Mitochondrial Morphology Evaluated by Transmission Electron Microscopy

After incubation with 1 µM vemurafenib, the SK-MEL-147 cells were prepared for transmission electron microscopy, as previously described [20]. The images were captured using the transmission electron microscope Jeol 1200 EXII (JEOL Brazil Scientific Instruments, Sao Paulo, Brazil) at the Federal University of Sao Paulo (UNIFESP).

### 4.10. Drug Combination

The CHOU–TALALAY method was used to assess synergism, antagonism, and the additive effects of the drug combinations. The data from concentration–response curves of each drug and the combination were obtained from MTT assays. The calculations to obtain the combination index (CI) were carried out using CompuSyn software v.1, and the formula is CI = (D)1/(Dx)1 + (D)2/(Dx)2, where D represents the single dose that produces a given effect and DX represents the combined dose that produces a given effect [37].

### 4.11. Bioinformatics

Raw RNA sequencing data from normal (*n* = 3) and melanoma (*n* = 3) samples presenting NRAS-type mutations were obtained from a previous study [36]. Replicate data were de novo assembled into a unified transcriptome using Trinity v2.15.1 software [81]. Then, cleaned RNA-Seq reads were aligned to the contigs generated by Trinity using the Bowtie2 v2.5 [82] software. Gene and isoform expression levels were quantified from the mapped data using RSEM v.1.3.3 [83]. Subsequently, the generated expression count tables were used as input for the differential expression (DE) analysis using the edgeR v.3.36.0 [84] package in R v.4.1.2, focusing on identifying isoforms with significant DE between the normal and melanoma samples. The results were based on FPKM (Fragments per Kilobase of Mapped Reads) values and visualized as bar graphs.

### 4.12. Statistical Analyses

All values were obtained from at least three independent experiments run in triplicate. The data were expressed as mean ± SEM, and statistical analyses were performed by one-way analysis of variance (ANOVA), followed by Tukey’s post hoc test, or by unpaired (two-tailed) Student’s *t*-test. Significance was defined as * *p* < 0.05, ** *p* < 0.01, *** *p* < 0.001, and **** *p* < 0.0001.

## 5. Conclusions

The data presented confirmed the paradoxical activation of the MAPK/ERK pathway. Unlike *BRAF*-mutated cells, *NRAS*-mutant cells did not exhibit mitochondrial hyperfused patterns under vemurafenib exposure, prevailing smaller and rounded mitochondria. Modulation fission using low concentrations of DRP1-inhibitor mdivi-1 sensitized the cells to vemurafenib and prevented paradoxical activation, a key resistance mechanism. We also found that bioenergetics disruption also sensitizes *NRAS* mutants to vemurafenib. Our findings clarify the molecular mechanisms of vemurafenib-induced MAPK/ERK pathway activation in NRAS^Q61R^ melanoma and demonstrate the potential of targeting mitochondria for combination chemotherapy.

## Figures and Tables

**Table 1:** Effect of vemurafenib and mdivi-1 interactions in the viability of NRAS^Q61R^ melanoma cells.

Time (h)	Vemurafenib (µM)	Mdivi-1 (µM)	CI
24	1.0	1.0	0.0219300 *
48	1.0	1.0	0.0000216 *
72	1.0	1.0	0.0569500 *

* The calculations were performed using the CHOU-TALALAY method with CompuSyn software v.1. Synergistic combinations have a combination index (CI) < 1.

**Table 2:** Effect of vemurafenib interaction with mitochondrial energetic modulators in the NRAS^Q61R^ SK-MEL-147 melanoma cell line.

Time (h)	Vemurafenib (µM)	CCCP (nM)	CI
4	1.0	25	0.08958 *
48	1.0	25	0.00290 *
72	1.0	25	0.20948 *
**Time (h)**	**Vemurafenib (µM)**	**Antimycin A (nM)**	**CI**
24	1.0	25	3.5060700 *
48	1.0	25	0.0000448 *
72	1.0	25	0.1414900 *
**Time (h)**	**Vemurafenib (µM)**	**Rotenone (nM)**	**CI**
24	1.0	25	6.87541 *
48	1.0	25	0.46380 *
72	1.0	25	0.81222 *

* Calculations performed using the CHOU-TALALAY method with CompuSyn software v.1. Synergistic combinations have a combination index (CI) < 1.

## Data Availability

The data sets generated and/or analyzed during the current study are available from the corresponding author upon reasonable request.

## Abbreviations

The following abbreviations are used in this manuscript:

*BRAF*	Rapidly Accelerated Fibrosarcoma B-type
*NRAS*	Neuroblastoma RAS Viral Oncogene Homolog
MAPK	Mitogen-Activated Protein Kinase
ERK	Extracellular Signal-Regulated Kinase
MEK	Mitogen-activated Extracellular Signal-regulated Kinase
FDA	Food and Drug Administration
DRP1	Dynamin-Related Protein 1
GTP	Guanosine Triphosphate
GDP	Guanosine Diphosphate
DMSO	Dimethyl Sulfoxide
Vem	Vemurafenib
MTT	3-(4,5-dimethyl-2-thiazolyl)-2,5-diphenyl-2H-tetrazolium Bromide
FITC	Fluorescein Isothiocyanate
AN	Annexin
PI	Propidium Iodide
MFN1	Mitofusin 1
MFN2	Mitofusin 2
OPA1	Optic Atrophy 1
Mdivi-1	Mitochondrial Division Inhibitor 1
CI	Combination Index
CCCP	Carbonyl Cyanide 3-chlorophenylhydrazone
AA	Antimycin A
DE	Differential Expression
*DNML1*	Dynamin-Like Protein 1
DUSP4	Dual Specificity Phosphatase 4
DUSP6	Dual Specificity Phosphatase 6
ROS	Reactive Oxygen Species
PRDX2	Peroxiredoxin 2
OXPHOS	Oxidative Phosphorylation
AMPK	AMP-Activated Protein Kinase
AICAR	5-aminoimidazole-4-carboxamide-ribonucleoside
DMEM	Dulbecco’s Modified Eagle’s medium
PMSF	Phenylmethylsulfonyl Fluoride
SDS-PAGE	Sodium Dodecyl Sulfate–polyacrylamide Gel Electrophoresis
HRP	Horseradish Peroxidase
ANOVA	One-way Analysis of Variance
